# Geographic Disparities in Domestic Pig Population Exposure to Ebola Viruses, Guinea, 2017–2019

**DOI:** 10.3201/eid3004.231034

**Published:** 2024-04

**Authors:** Solène Grayo, Alimou Camara, Bakary Doukouré, Isabelle Ellis, Cécile Troupin, Kerstin Fischer, Jessica Vanhomwegen, Michael White, Martin H. Groschup, Sandra Diederich, Noël Tordo

**Affiliations:** Institut Pasteur de Guinée, Conakry, Guinea (S. Grayo, B. Doukouré, I. Ellis, N. Tordo);; Centre de Recherche en Virologie, Laboratoire des Fièvres Hémorragiques Virales de Guinée, Conakry (A. Camara);; Institut Pasteur du Laos, Vientiane, Laos (C. Troupin);; Friedrich-Loeffler-Institut, Standort Insel Riems, Germany (K. Fischer, M.H. Groschup, S. Diederich);; Institut Pasteur, Paris, France (J. Vanhomwegen, M. White)

**Keywords:** viruses, serology, Ebola virus, swine, pigs, Guinea, West Africa

## Abstract

Although pigs are naturally susceptible to Reston virus and experimentally to Ebola virus (EBOV), their role in *Orthoebolavirus* ecology remains unknown. We tested 888 serum samples collected from pigs in Guinea during 2017–2019 (between the 2013–16 epidemic and its resurgence in 2021) by indirect ELISA against the EBOV nucleoprotein. We identified 2 hotspots of possible pig exposure by IgG titer levels: the northern coast had 48.7% of positive serum samples (37/76), and Forest Guinea, bordering Sierra Leone and Liberia, where the virus emerged and reemerged, had 50% of positive serum samples (98/196). The multitarget Luminex approach confirms ELISA results against Ebola nucleoprotein and highlights cross-reactivities to glycoprotein of EBOV, Reston virus, and Bundibugyo virus. Those results are consistent with previous observations of the circulation of *Orthoebolavirus* species in pig farming regions in Sierra Leone and Ghana, suggesting potential risk for Ebola virus disease in humans, especially in Forest Guinea.

After the original detection of Zaire Ebola virus (EBOV; species *Orthoebolavirus zairense*) in Yambuku, Democratic Republic of Congo (DRC), in 1976, an additional 5 species have been identified: *Orthoebolavirus sudanense* (Sudan virus [SUDV]) in Sudan in 1976, *Orthoebolavirus restonense* (Reston virus [RESTV]) in the United States in 1989, *Orthoebolavirus taiense* (Tai Forest virus [TAFV]) in Cote d’Ivoire in 1994, *Orthoebolavirus bundibugyoense* (Bundibugyo virus [BDBV] in Uganda in 2007, and *Orthoebolavirus bombaliense* (Bombali virus [BOMV]) in Sierra Leone in 2016 (*1*). EBOV, SUDV, TAFV, and BDBV have caused Ebola virus disease (EVD) in humans; EBOV has the highest case-fatality rates of up to 90% and is responsible for most EVD outbreaks in sub-Saharan Africa (*2*,*3*). Until 2013, EVD was thought to be confined to Central Africa (Gabon, DRC, Congo, Uganda), and epidemics were limited to hundreds of cases at the most and lasted less than a few months (*2*). In West Africa, a sporadic emergence of TAFV in chimpanzees of the Taï forest in Cote d’Ivoire caused a single nonlethal human case in 1994 (*4*,*5*); after that, the worst EBOV epidemic occurred in Guinea, Sierra Leone, and Liberia during 2013–2016 (*6*) and resurged in Guinea in 2021 (*7*). The same phenomena of expansion to West Africa can currently be observed with Marburg disease, which was detected sporadically in Guinea in 2021 (*8*), Ghana in 2022 (*9*,*10*), and, more recently, Equatorial Guinea (*11*) and Tanzania, whereas former outbreaks were limited to Central-South Africa (Angola, DRC, Kenya, South Africa, Uganda, Zimbabwe) (*12*). The spatial expansion of hemorrhagic fevers caused by filoviruses in West Africa spotlights viral adaptation to a new environment and calls into question the role of wildlife and livestock in ebolavirus ecology (*13*–*15*). On the basis of molecular and serologic traces of filovirus infection, bats are the most commonly suspected wildlife reservoirs (*16*–*22*). However, only MARV has been successfully isolated directly from Egyptian fruit bats (*Rousettus aegyptiacus*) (*23*). Other isolates from bats are still pending. Even by combining wildlife surveys and molecular screening of bat and environmental samples, no convincing evidence for a bat origin of the West Africa epidemic was confirmed (*15*).

Sporadic human Ebola infections through contact with chimpanzees, gorillas, duikers, and other wild mammals have been reported, but the role played by domestic animals and livestock as intermediate hosts in the maintenance, amplification, and transmission to humans has been poorly explored (*13*,*14*,*24*–*27*). Pigs can act as intermediate amplifying hosts for endemic or emerging viruses, leading to disease outbreaks in humans (e.g., Nipah virus in Malaysia and Singapore in the late 1990s) (*28*). After experimental infection, EBOV causes respiratory clinical symptoms in piglets and oronasal shedding and transmission to cohoused piglets and nonhuman primates (*25*,*29*). Pigs are also naturally susceptible to RESTV, as demonstrated in the Philippines in 2008 (*30*), and RESTV‐specific antibodies have been found in healthy workers from pig farms, suggesting possible transmission from pigs to humans (*30*,*31*). RESTV is thus presumed not pathogenic for humans, but its pathogenicity for pigs remains unclear (*32*–*34*).

In recent years, serologic evidence of circulation of various Ebola virus species in pigs has been found in countries in West and East Africa. Studies during EVD outbreaks in Uganda (*35*), Sierra Leone (*36*), and Guinea (*37*) demonstrated some cross-reactivity against nucleoproteins (NPs) of several species, such as EBOV-NP, RESTV-NP, and SUDV-NP, suggesting possible circulation of different species. In Ghana, a country with no known EVD outbreak, 5/139 pig serum samples reacted against different glycoproteins (GPs) of EBOVs: 2 against TAFV-GP, 1 against EBOV-GP, 1 against RESTV-GP, and 1 against Lloviu virus GP (LLOV-GP) (*38*). In Guinea, which was part of the large West Africa outbreak of EVD in 2013, 19/308 pig serum samples (6.2%) collected in the Conakry area were seroreactive to EBOV-NP by ELISA. Among those 19 samples, 13 were confirmed positive by Western blot analysis against EBOV-NP, 4 cross-reacted against SUDV-NP, and 13 cross-reacted against RESTV-NP (*38*). That preliminary investigation in Conakry could be biased by the large pig trade attracting animals from other regions to the capital. This study has been extended to most pig farming areas in the different ecosystems of Guinea, in particular Forest Guinea in the southeast of the country, where the EVD outbreak started and where different species, such as BOMBV, have been detected (*39*). The study relies on 888 pig serum samples collected during 2017–2019, between the large 2013–2016 EVD outbreak and its 2021 resurgence. We screened those serum samples using a previously established in-house ELISA against EBOV-NP and also using the multiplex microsphere immunoassay (MMIA) based on the Luminex technology (Luminex, https://www.diasorin.com), which enabled evaluation of serum reactivity against several antigens of several species of *Orthoebolavirus* in parallel. Overall, this approach enabled us to estimate potential hotspots of pig exposure to multiple *Orthoebolavirus* species in different regions of Guinea.

## Methods

### Study Area, Ethical Approval, and Sampling

During October 2017–June 2019, we collected 888 domestic pig serum samples from different pigsties covering 7 of the 8 administrative regions of Guinea (Appendix Figure). The population of Guinea is 85% Muslim, so pig farming is mostly found in the Christian region of Forest Guinea, where we selected 2 sites: NZérékoré, a large city in Forest Guinea with suitable sanitation and access to electricity, and Koulé, a small rural village with practices of open defecation and the close proximity of pigsties and shared water resources. Other pigsties were found in the mixed population area along the Conakry-Kindia axis and on the northern seashore, where the bauxite mining industry attracts foreigners. No significant pig raising is performed in the large northeastern area. 

For each site, we recorded GPS (Global Positioning System) coordinates and noted ecologic conditions of the pigsties (open air or closed), as well as the sex, age, and weight of each sampled animal. We excluded pregnant or lactating females and piglets <3 months of age from the study as specified in the protocol (no. 040/CNERS/17) agreed to by the Comité National d’Éthique pour la Recherche en Santé of Guinea. We collected blood from the precaval vein of randomly selected pigs by using sterile vacutainer tubes without anticoagulant and centrifuged at 2,000 × *g* for 20 minutes. We stored the resulting serum at −20°C in a cold container until it was transported and stored at –80°C in the Biobank of the Institut Pasteur de Guinée.

### Indirect IgG ELISA based on *Escherichia coli*–Produced EBOV Nucleoprotein

We evaluated the seroreactivity of pig serum by an in-house indirect IgG ELISA assay targeting the EBOV NP produced in *Escherichia coli* (*36*,*38*). Serum samples were heat inactivated for 30 minutes at 56°C, then diluted at 1:100 and analyzed according to the ELISA protocol (*36*), including negative and positive serum controls, as well as the *E. coli* extract to evaluate nonspecific binding. We monitored the optical density (OD) at 405 nm in a Multiskan FC Microplate Photometer (ThermoFisher Scientific, https://ww.thermofisher.com). We first validated the assay to ensure the positive control serum (from a pig immunized with EBOV-like particles) reached a predetermined OD_405_ range of 0.7–0.9. A cut‐off OD_405_ value of 0.19 with an inconclusive window (0.16–0.19; i.e., <0.16 = negative; 0.16–0.19 = inconclusive; >0.19 = positive) was established as the mean value of corrected ODs plus 3 SDs, as in Fischer et al. (*38*). We tested serum samples >2× to provide a final conclusion in terms of reactivity against EBOV-NP (EBOV strain Mayinga, Zaire, 1976) (*36*).

### Multiplex Microsphere Immunoassay with Filovirus 4-Plex

We used a previously published MMIA adapted for pig serum (*21*,*40*) to determine the presence of IgG against different antigens of orthoebolaviruses. Each of 4 color-coded magnetic bead sets (Bio-Plex ProTM Magnetic COOH Beads; Bio-Rad, https://www.bio-rad.com) was coupled at room temperature to a specific *Orthoebolavirus* antigen through carboxylate amine bonds using the Bio-Plex Amine Coupling Kit (Bio-Rad). The 4 antigens were EBOV-NP produced in *E. coli* and EBOV-GP, BDBV-GP (both Sino Biological, https://www.sinobiological.com), and RESTV-GP (IBT Bioservices, https://www.ibtbioservices.com) produced in Sf9 cells. EBOV-NP corresponds to EBOV strain Mayinga, 1976 (Gentaur, #544-MBS1206629), EBOV-GP to EBOV strain Mayinga, 1976 (Sino Biologic, #40304-V08B1), BDBV-GP to BDBV strain Uganda, 2007 (Sino Biologic, #40368-V08B), and RESTV-GP to RESTV strain Philippines, 1996 (Gentaur, #494–0504–015). 

We diluted heat-inactivated serum samples at 1:400 in 50 μL of assay buffer (phosphate-buffered saline, 1% bovine serum albumin solution, 0.05% Tween-20) mixed with the antigen-coated bead sets (≈1,250 beads of each type) and placed them in the Bio-Plex Pro flat-bottom well of the MIA plate (Bio-Rad) and protected them from light. After 30 min incubation at room temperature with shaking at 700 rpm, we washed the plate 3 times with the washing solution (phosphate-buffered saline, 0.05% Tween-20). After washing, we added 50 μL of a secondary biotinylated goat antiswine IgG (Jackson ImmunoResearch, https://www.jacksonimmuno.com) at 4 μg/mL in assay buffer to each well and incubated at room temperature on an orbital shaker for 30 minutes at 700 rpm in the dark. After washing 3 times, we incubated the beads for 10 minutes at 700 rpm in the dark with 50 μL of Streptavidin-R-Phycoerythrin (ThermoFisher Scientific) diluted to 2 μg/mL in assay buffer. After 3 additional washing steps, we resuspended the beads in 100 μL of xMAP assay buffer (Bio-Rad) and agitated for 2 min at 1,100 rpm in the dark. We performed measurements using a Magpix instrument (Luminex). We measured fluorescence intensity and bead color coding by dual lasers at 2 different wavelengths: 635 nm to identify the microsphere particle and 532 nm to excite the Streptavidin-R-Phycoerythrin reporter dye. At least 100 events were read for each bead set, and binding events were displayed as mean fluorescence intensities (MFI). For each sample, we calculated MFI from >50 beads bearing the same antigen.

### Quantitative Reverse Transcription PCR

We performed EBOV genome detection in the serum of pigs from sites with significantly higher seroreactivity. We carried out nucleic acid extraction using an ID Gene Mag Fast Extraction Kit and IDEAL 32 extraction robot (Innovative Diagnostics, https://www.innovativediagnostics.com). We performed EBOV genome detection on 10 μL of extracted RNA using the RealStar Filovirus Screen quantitative reverse transcription PCR Kit 1.0 (Altona Diagnostics, https://altona-diagnostics.com), according to manufacturer protocols. We interpreted samples with cycle threshold (Ct) >40 and a positive internal control as EBOV-negative and samples with Ct >0 and <40 as EBOV-positive.

### Statistical Analysis

We used the Pearson χ^2^ test to determine whether there was any association between EBOV serology results (i.e., number of positive pig serum samples) and 3 variables (sex, age, and sampling site) by assuming that the grouping variable and outcome are independent. Then, we estimated the strength of the association from a univariate logistic regression model using only dichotomous ELISA status of pig serum (1 = positive; 0 = negative) and excluding inconclusive serum samples from the ELISA data (n = 45). We built the generalized linear models under the form: “OD (numeric) ~ term (linear predictor for response)” (“ELISA ~ sex,” or “ELISA ~ age,” or “ELISA ~ sampling site”) to obtain the odds ratio (OR) of positive versus negative pigs in function of sex, age, and site. The OR’s interpretation of the classes is made relative to the reference (Table). In this study, the reference classes were female, 3–6 years old, and Conakry. We considered p values of <0.05 to be statistically significant, meaning the true odds ratio of the overall population was within range of the 95% CI (1 – α). We performed all data analyses using RStudio version 2022.07.2 and plotted maps using SimpleMaps (https://simplemaps.com).

**Table:** Seroreactivity to EBOV-NP IgG and risk factors of exposure to EBOV in domestic pig population, Guinea, 2017–2019*

Variable	Class	Total	EBOV-NP IgG seroreactivity				Risk factors	
			Positive	Negative	Inconclusive		OR (95% CI)	p value
Sex	F†	440	114 (25.91)	308 (70)	18 (4.09)		NA	NA
	M	448	109 (24.33)	312 (69.64)	27 (6.03)		0.94 (0.69–1.28)	0.712
Age, mo	3–6†	490	121 (24.69)	338 (68.98)	31 (6.33)		NA	NA
	7–12	282	83 (29.43)	188 (66.67)	11 (3.90)		1.23 (0.89–1.72)	0.215
	>13	116	19 (16.38)	94 (81.03)	3 (2.59)		0.56 (0.33–0.96)	<0.05
Sites	Boké	29	11 (37.93)	12 (41.38)	6 (20.69)		11.23 (3.25–46.58)	<0.001
	Boffa	47	26 (55.32)	21 (44.68)	0		15.17 (5.16–56.35)	<0.001
	Dubreka	85	5 (5.88)	75 (88.24)	5 (5.88)		0.82 (0.21–3.44)	0.77
	Conakry†	53	4 (7.55)	49 (92.45)	0		NA	NA
	Coyah	169	18 (10.65)	148 (87.57)	3 (1.78)		1.49 (0.52–5.34)	0.49
	Forecariah	41	0	41 (100)	0		≈0	0.98
	Kindia	84	27 (32.14)	49 (58.33)	8 (9.52)		6.75 (2.42–24.11)	<0.001
	Dalaba	15	1 (6.67)	14 (93.33)	0		0.88 (0.04–6.53)	0.91
	Kissidougou	94	7 (7.45)	85 (90.43)	2 (2.13)		1.01 (0.29–4.01)	0.99
	Guéckédou	75	26 (34.67)	42 (56)	7 (9.33)		7.58 (2.69–27.26)	<0.001
	Koulé	43	36 (83.72)	5 (11.63)	2 (4.65)		88.20 (24.79–408.7)	<0.001
	Nzérékoré	153	62 (40.52)	79 (51.63)	12 (7.84)		9.61 (3.67–33.09)	<0.001
Total		888	223 (25.11)	620 (69.82)	45 (5.07)			

*Values are no. (%) except as indicated. EBOV, Zaire Ebola virus; NA, not applicable; OR, odds ratio. †Indicates the reference level for 3 categorical variables (sex, age, and region).

**Figure 1:**
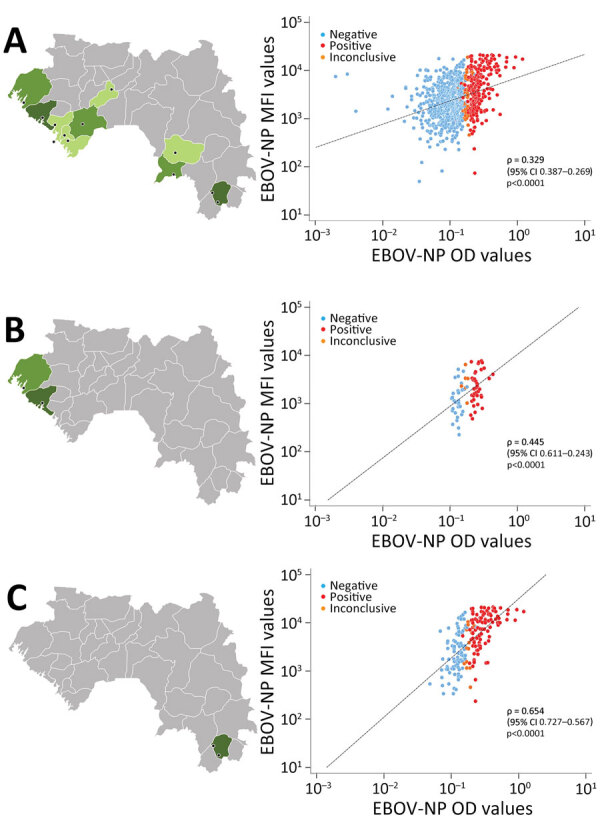
Correlation of indirect ELISA and multiplex microsphere immunoassay for EBOV-NP in study of geographic disparity in domestic pig population exposure to Ebola viruses, Guinea, 2017–2019. Scatter plots of MFI values obtained by multiplex microsphere immunoassay and OD values at 405 nm (OD 405) obtained by ELISA for pig serum samples are shown for all testing sites in Guinea (n = 882) (A), the northern coast (n = 75) (B), and the Forest Guinea (n = 196) (C). Black dashed lines represent reduced major axis lines; ρ indicates Spearman coefficient of rank correlation. Black dots on map indicate study location as detailed in Figure 3. EBOV, Zaire Ebola virus; MFI, mean fluorescence intensities; NP, nucleoprotein; OD, optical density.

**Figure 2:**
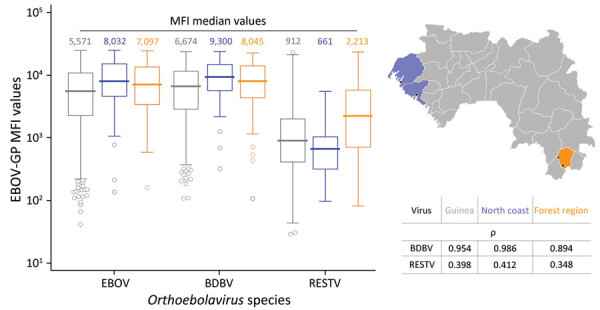
Pig serum samples tested by multiplex microsphere immunoassay against GP recombinant proteins of different *Orthoebolavirus* species in study of geographic disparity in domestic pig population exposure to Ebola viruses, Guinea, 2017–2019. MFI values of pig serum against EBOV, BDBV, and RESTV-GPs are shown for all testing sites in Guinea (gray), the northern coast (blue), and the Forest Guinea (orange), corresponding to locations on the map at right. Table outlines the Spearman coefficient of rank correlation (ρ) values between the species EBOV and each of the 2 species BDBV and RESTV. The associated p value was <0.0001 in all cases. Black dots on map indicate study location as detailed in Figure 3. BDBV, Bundibugyo virus; EBOV, Zaire Ebola virus; GP, glycoprotein; MFI, mean fluorescence intensities; RESTV, Reston virus.

We calculated the median MFI signal for each antigen and represented it by a thick horizontal bar in the graphs. We determined the relationship between the 2 variables by estimating the Spearman coefficient of rank correlation (ρ: number between −1 and 1; no normal distribution of variables) with a 95% CI and represented it graphically by a scatter diagram (Figure 1). A p value <0.05 means the correlation coefficient is statistically significant. We constructed graphs (Figures 1, 2) and performed statistical analyses using MedCalc Statistical Software version 20.215 for Windows (MedCalc Software, https://www.medcalc.org).

## Results

### In-House ELISA to Recognize EBOV-NP in Pig Serum 

Of the 888 pig serum samples tested by EBOV-NP protein ELISA, a similar number were from male (n = 448) and female (n = 440) animals (Table). A total of 223 samples (25.11%) were ELISA-positive, and no significant difference was found between sexes: 114/440 (25.91%) female and 109/448 (24.33%) male (χ^2^ = 1.866; p>0.05). The absence of an association with gender was supported by the estimated OR 0.94 (95% CI 0.69–1.28; p = 0.3934). The mean age of the pigs was 8 months and median was 6 months. In the 3–6-month age group, 24.69% of serum samples were reactive; 29.43% of serum samples in the 7–12-month age class were reactive, and 16.38% of serum samples in the >13 months age class were reactive. The estimated OR between age classes 3–6 months and 7–12 months was 1.2 (95% CI 0.89−1.72), which is not significant (p = 0.215).

**Figure 3:**
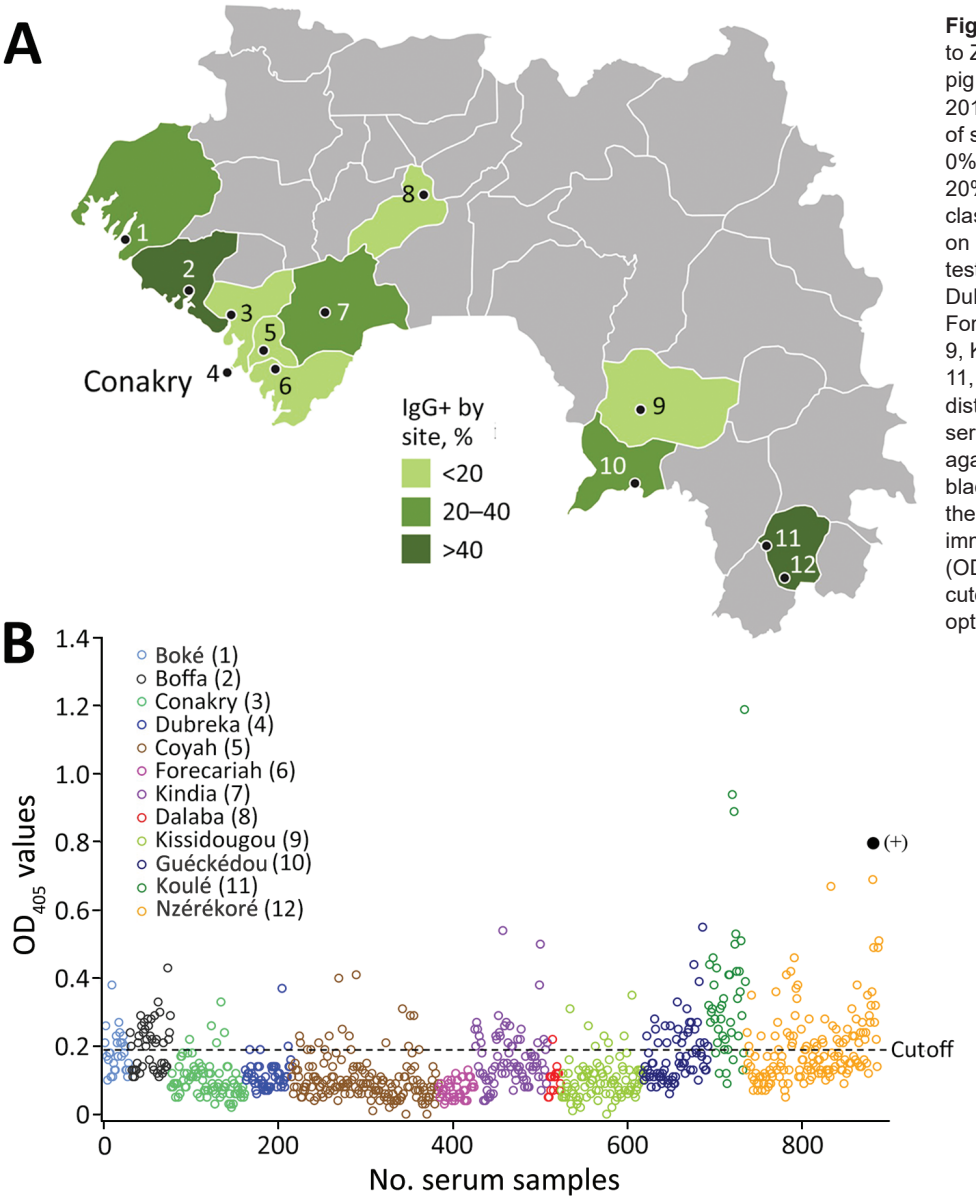
Disparity in seroreactivity to Zaire Ebola virus (EBOV) in pig farming regions, Guinea, 2017–2019. A) Spatial distribution of seroreactivity: lower class, 0%–20% seroreactivity; middle class, 20%–40% seroreactivity; and higher class, >40% seroreactivity. Numbers on map and in panel B key indicate testing sites: 1, Boké; 2, Boffa; 3, Dubreka; 4, Conakry; 5, Coyah; 6, Forecariah; 7, Kindia; 8, Dalaba; 9, Kissidougou; 10, Guéckédou; 11, Koulé; 12, Nzérékoré. B) Plot distribution of OD values of the 888 serum samples tested by ELISA against EBOV nucleoprotein. Solid black circle at right top represents the OD value of the serum from a pig immunized with EBOV-like particles (OD 0.8). Dashed line represents the cutoff value of the assay (0.19). OD, optical density.

Seroreactivity for 12 collection sites showed substantial variations, ranging from 0% to 83.7% (Table). Overall, the lower class (0%–20%) of seroreactivity included the greater Conakry area (Coyah 10.7%, Conakry 7.6%, Dubreka 5.9%, and Forecariah 0%) and 2 more distant sites, in middle Guinea (Dalaba 6.7%; only 15 serum samples collected) and Kissidougou (7.5%) in the southern part of the country (Figure 3, panel A). In that group, no site demonstrated significantly different risk for seroreactivity than Conakry (Dubreka OR = 0.82, Coyah = 0.49, Forecariah = ≈0, Dalaba = 0.88, and Kissidougou = 1.01; p>0.05). That finding was in contrast to the second class of seroreactivity (20%–40%), which included Kindia (32.1%), a main stopover for livestock drivers before the capital area, Boké (37.9%) in the northwest, and Guéckédou (34.6%) in the southeast; OR values were 6.8 (Kindria), 7.6 (Boké), and 11.2 (Guéckédou), indicating significantly higher seroreactivity than for Conakry (p<0.001). The group demonstrating the highest seroreactivity rate (>40%), corresponded to the furthest sites from Conakry: Boffa (55.32%) in the northwest part of Guinea, Nzérékoré (40.5%), and Koulé (83.7%) in the southeast part of Guinea. The estimated OR values were significant for all those sites (p<0.001), reaching 88.2 for Koulé in Forest Guinea. The geographic contrast in terms of seroreactivity was also mirrored in the distribution of OD values; the peak corresponded to southeastern sites and the trough to the Conakry area (Figure 3, panel B). The OD values were particularity high in Forest Guinea, where some pig serum samples exceeded the positive control OD value, suggesting a seroreactivity to EBOV or to another EBOV species by cross-reactivity for the pig population in this region. 

We tried to detect EBOV genome in 431 serum samples from the sites with significantly higher seroreactivity using the RealStar Filovirus Screen RT-PCR kit 1.0 (Altona Diagnostics). No positive serum was obtained from Boké (n = 29), Boffa (n = 47), Kindia (n = 84), Guéckédou (n = 75), Koulé (n = 43), or Nzérékoré (n = 153).

### MMIA

**Figure 4:**
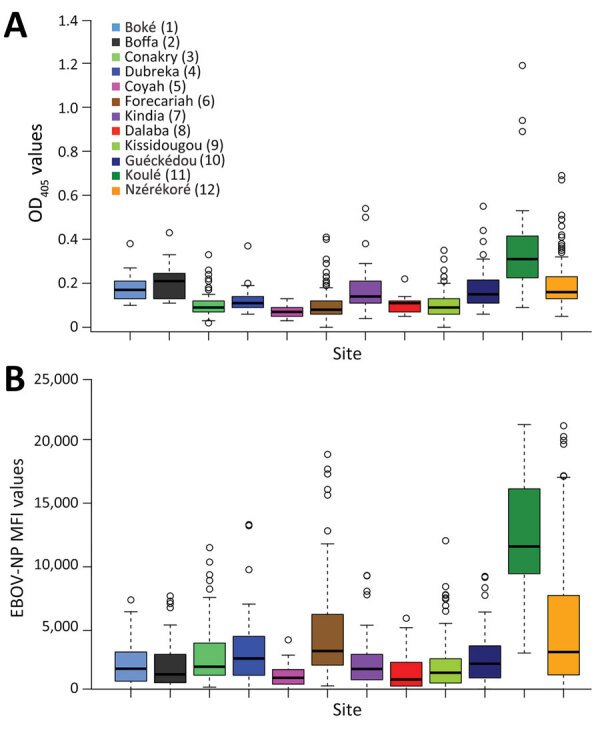
Comparison between results of indirect ELISA and multiplex microsphere immunoassay for EBOV-NP in study of geographic disparity in domestic pig population exposure to Ebola viruses, Guinea, 2017–2019. A) Boxplot of OD values at 405 nm (OD_405_) obtained by ELISA by site (n = 888 pig serum samples). B) Boxplot of MFI values obtained by multiplex microsphere immunoassay (n = 882 pig serum samples). Locations in key correspond to locations on map in Figure 3. EBOV, Zaire Ebola virus; MFI, mean fluorescence intensities; NP, nucleoprotein; OD, optical density.

We also tested pig serum samples using MMIA technology at 1:400 dilution in comparison to the ELISA technology. Under boxplot presentation, the ELISA results expressed in OD values (Figure 4, panel A) and the MMIA results expressed in MFI values distribution (Figure 4, panel B) showed the same global mean tendency across the collection sites, indicating a good correlation between the 2 technologies. Of note, we observed high reactivity in Forest Guinea, particularly in Koulé.

To better evaluate the correlation between ELISA (OD values) and MMIA (MFI values), we constructed scatter plots using the data of all testing sites in Guinea (Figure 1, panel A), those of the northern coast in the Maritime Guinea region (Boké, Boffa) (Figure 1, panel B), and data from Forest Guinea (Nzérékoré and Koulé) (Figure 1, panel C). When looking at all testing sites together (Figure 1, panel A), the 3 subgroups (negative, positive, and inconclusive) defined by the ELISA assay were not distinguishable by MFI values; the regression line tended to horizontal, and the global correlation coefficient was weak (n = 882, ρ = 0.329). The northern coast panel (Figure 1, panel B) showed a better correlation between both immunoassays (n = 75, ρ = 0.445). Finally, the highest correlation, at ≈2 times more than all testing sites in Guinea, was observed in Forest Guinea (n = 196, ρ = 0.654), where more positive serum samples clustered on the top right and negative serum samples clustered on the bottom left.

To further evaluate the specificity of the pig serum, we performed a multiplex analysis against the GPs of 3 *Orthoebolavirus* species: EBOV, BDBV, and RESTV. We compared the GP MFI distributions of all sites in Guinea, the northern coast, and Forest Guinea (Figure 2). Independent of the sample size or location, the median values of EBOV-GP (MFI 5,571) and BDBV-GP (MFI 6,674) were relatively close, supporting cross-reactivity between the different GPs as outlined by their amino-acid sequence identity of 70% (*41*). The median values of the RESTV-GP (MFI 913) were lower according to its amino acid sequence identity of only 58% with EBOV-GP (*41*,*42*). In addition, the reactivity against RESTV-GP of samples from Forest Guinea was clearly and significant higher (MFI 2,213) than that of samples from the northern coast (MFI 661) (p<0.0001 by Kolmogorov-Smirnov test).

## Discussion 

This study not only reinforces findings from previous studies regarding exposure of pigs to *Orthoebolavirus* species in Central and West Africa (*36*,*37*) but also sheds new light on the definition of potential regions at risk in Guinea. Although previous work demonstrated seroreactivity to EBOV-NP in 6.2% (19/308) of pig serum samples in pigsties around Conakry (*38*), by expanding data collection to 888 pig serum samples across Guinea, we demonstrated an overall seroreactivity of 25% (221/888) (i.e., 4× higher). Our collection from 12 different sites in Guinea covered most of the country’s terrestrial ecosystems, from the ocean mangrove and swamp forest along the Atlantic littoral zone up to the evergreen and semideciduous rainforests in Forest Guinea (*43*). We only excluded the northeast part of Guinea from our investigation because of the lower rates of pig farming in that region. We observed geographic disparities in orthoebolavirus exposure in pigs, regardless of sex and age.

The 2 hotspots of EBOV exposure for pigs were observed in pigsties with roof and outside openings in rural regions. However, the hotspots differed in ecologic conditions: 1 was in the ocean mangrove on the North Coast and 1 was in the mountain highlands of Forest Guinea where biodiversity is high. That different ecosystem might influence the exposure of pigs to orthoebolaviruses and explain the higher OD values in Koulé in Forest Guinea using either ELISA or MMIA technology.

We found a significant correlation between the 2 technologies for EBOV-NP detection, which was especially high in Forest Guinea (ρ = ≈0.7). In addition, multiplexing using the more specific GP proteins allowed us to compare 3 *Orthoebolavirus* species. The MMIA assay showed similar reaction patterns against EBOV-GP and BDBV-GP independent of the sampling location (*44*,*45*). However, reactivity to RESTV-GP was clearly higher in the southeast in Forest Guinea and less reactive in the northwest coastal region. That result is consistent with a previous study in Sierra Leone in which a pig only reacted with RESTV-NP (*36*). This similar finding between neighboring countries where pigs live in a bush habitat with probable wildlife contact suggests exposure to a different unidentified *Orthoebolavirus* species with possible zoonotic or pathogenic potential. BOMV recently discovered in insectivorous bats in both countries could be suspected to be phylogenetically positioned between RESTV and EBOV (*22*,*46*). Its detection in Nzérékoré in March 2019 occurred just before our campaign in Forest Guinea in June 2019. It should be noted that no positive pig serum was detected by RT-PCR using the RealStar Filovirus Screen RT-PCR kit 1.0, which is, however, unable to detect BOMV.

Overall, our study further emphasizes the need to deepen monitoring in areas of high seroprevalence in pigs and further evaluate filovirus pathogenicity for pigs and human. It would be key to conduct a joint investigation in humans, particularly in populations at risk (e.g., farmers, veterinarians, slaughterhouse workers), and to provide information about the risks of consuming undercooked pork products, which would be helpful for many pathogens (hepatitis E virus, Nipah virus, influenza A virus). The limitations of free-range pig husbandry and open sanitation in villages might also be considered to avoid pig exposure to EBOV from human shedding. In this study, however, a direct link with EBOV circulating in humans during the 2014–16 epidemic is highly improbable because sampling began at the end of 2017 (i.e., 1.5 years after the end of the epidemic) and most pigs tested were <1 year of age. Finally, in the One Health context, exploring the relevance of the specific ecologic surrounding of ocean mangrove or rainy forest is key. Bats have long been considered the most likely suspects, but more attention must be paid to peridomestic micromammals, such as rodents, which could serve as links between the village where they find subsistence and wildlife in the forest. Pigs might be infected by their contaminated urine and feces. One experimental study has shown mutations associated with *Orthoebolavirus* adaptation to rodents (*47*). Investigating zoonotic *Orthoebolavirus* infection in rodents, as well as in pig populations at a local level to evaluate the potential risk of human exposure would be key. 

Appendix Additional information about geographic disparities in domestic pig population exposure to Ebola viruses, Guinea, 2017–2019.
