# Supplementary material for: Geographic Disparities in Domestic Pig Population Exposure to Ebola Viruses, Guinea, 2017–2019
Source: Emerg Infect Dis. 2024 Apr;30(4):681–90. doi: 10.3201/eid3004.231034 (PMC10977825; doi:10.3201/eid3004.231034)
Supplement: Appendix — Additional information about geographic disparities in domestic pig population exposure to Ebola viruses, Guinea, 2017–2019. [file 23-1034-Techapp-s1.pdf]

Article DOI: https://doi.org/10.3201/eid3004.231034

EID cannot ensure accessibility for supplementary materials supplied by authors. Readers who have difficulty accessing supplementary content should contact the authors for assistance.

Geographic Disparities in Domestic Pig Population Exposure to Ebola Viruses, Guinea, 2017–2019

Appendix

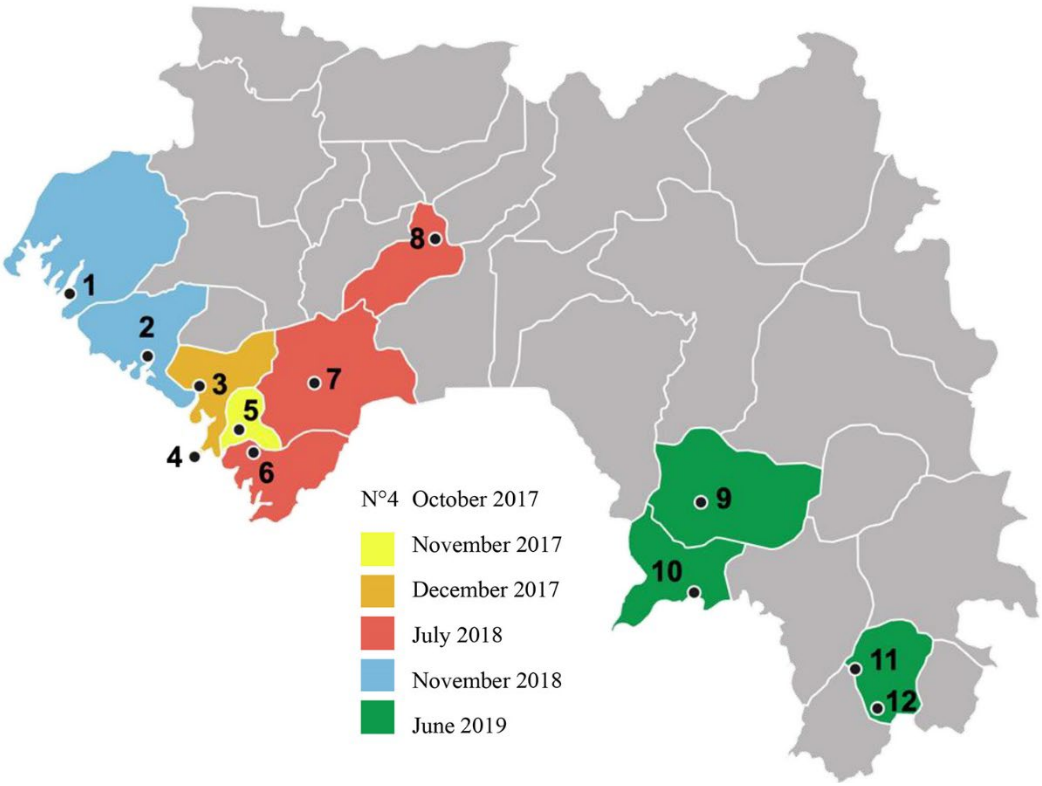

Appendix Figure. Sample collection of pig serum from 2017–2019 in various natural regions of Guinea. The pigs sampling came from a large livestock animal collection consisting of 6 campaigns performed between 2017 and 2019 as following: the capital, Conakry, the middle maritime region in yellow and orange, the lower maritime region and the near hinterland in red, the upper maritime region in blue, and the forest region in green.
